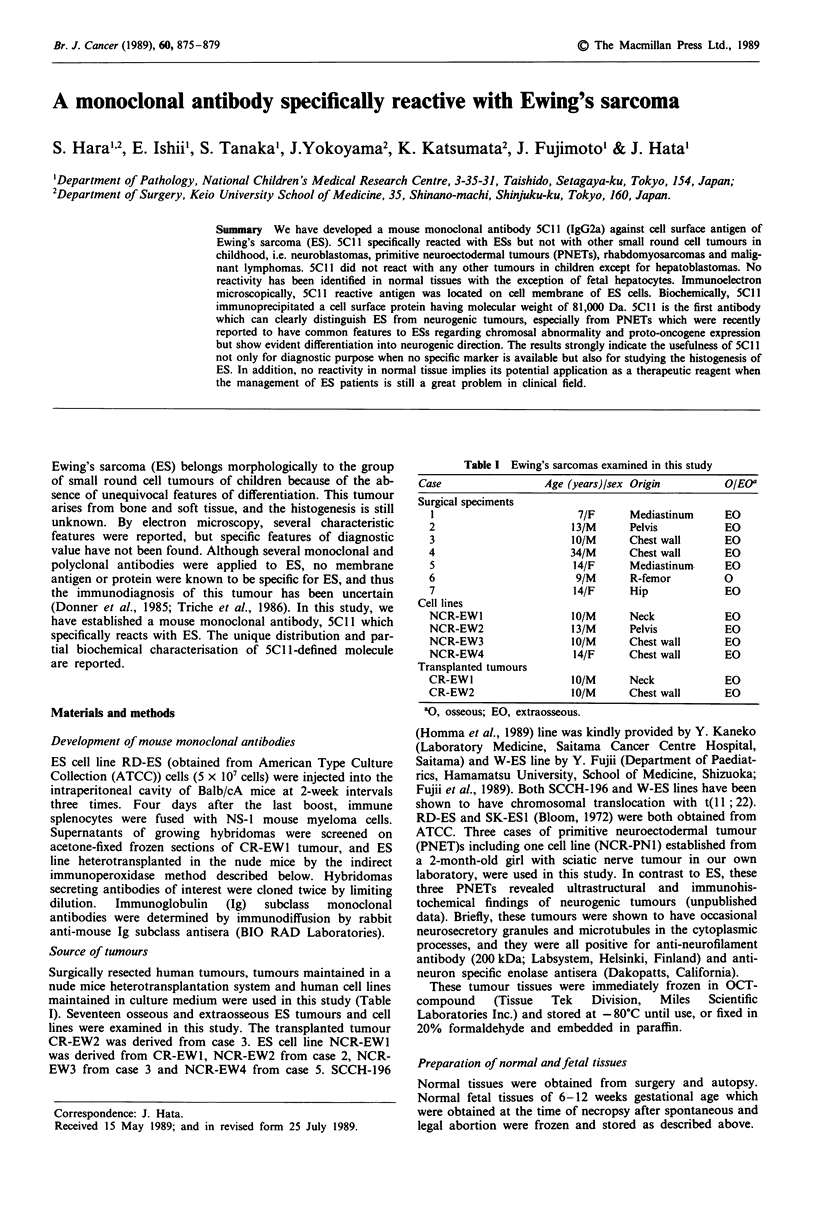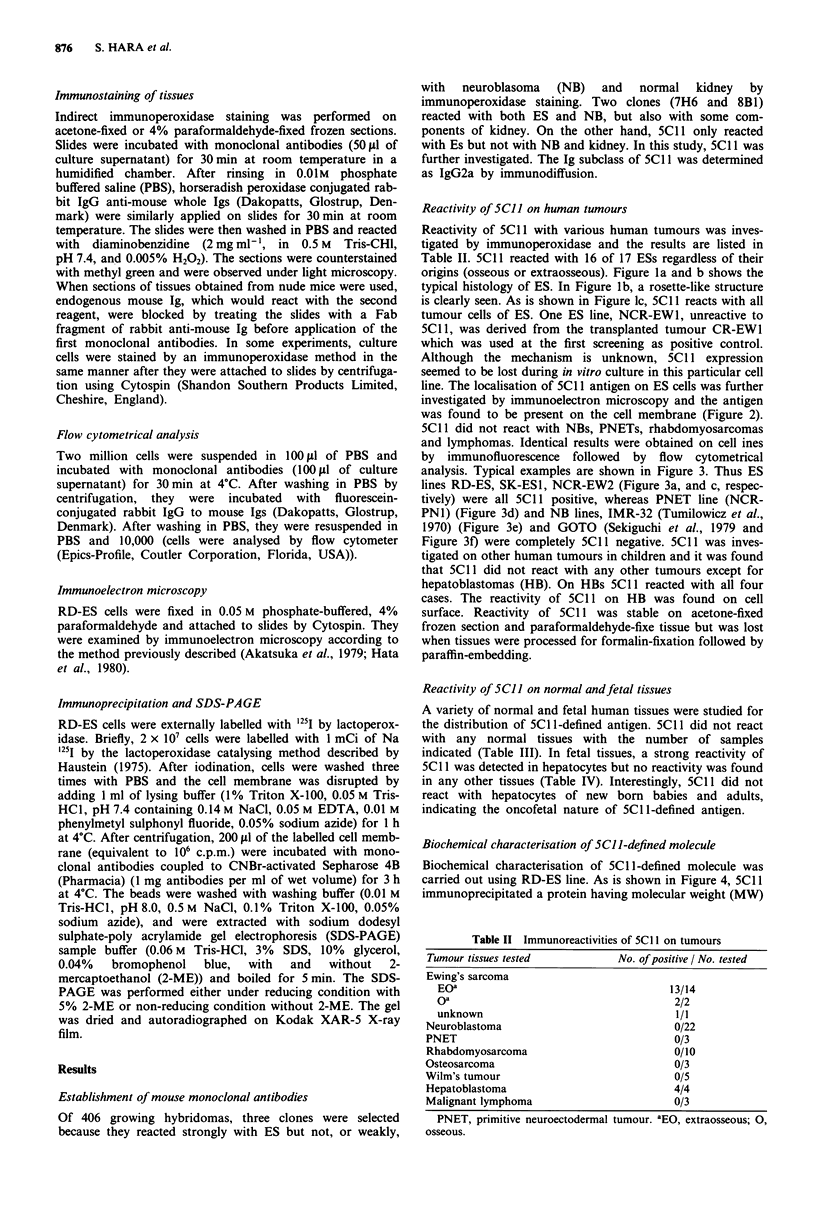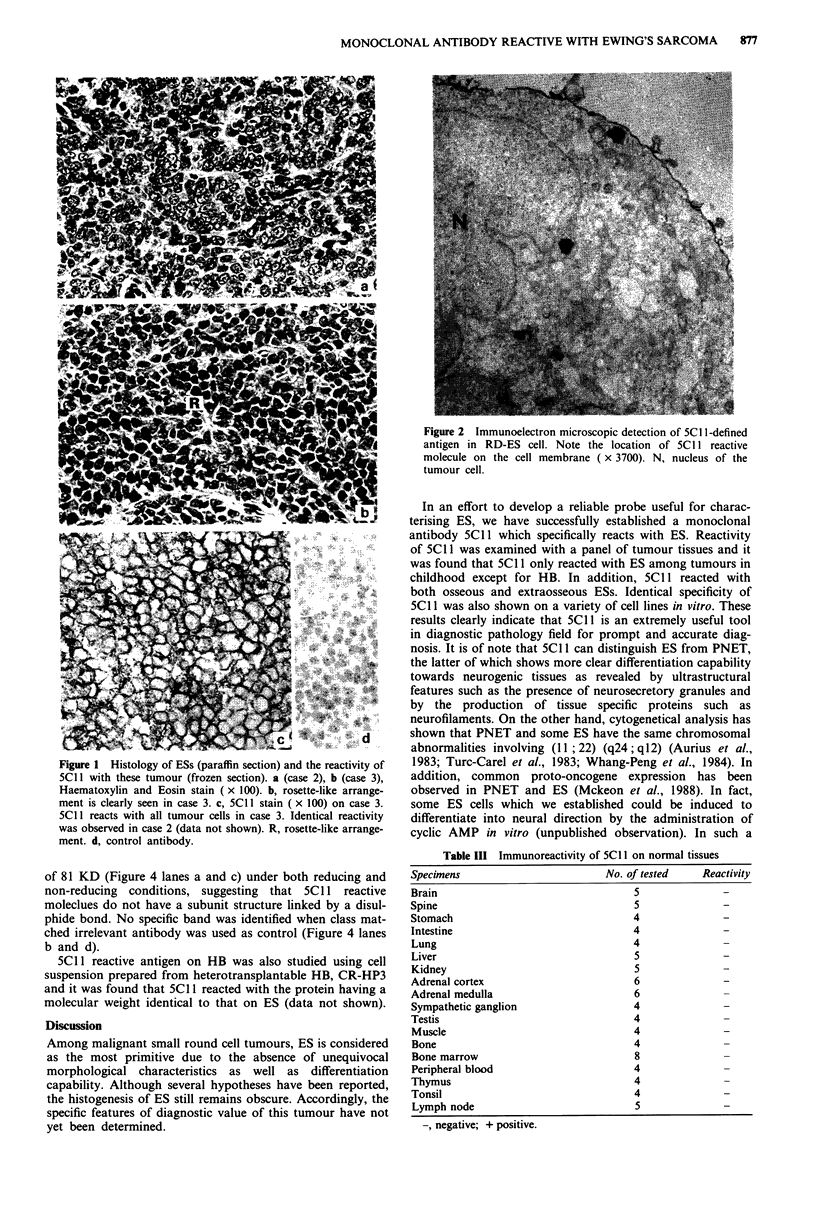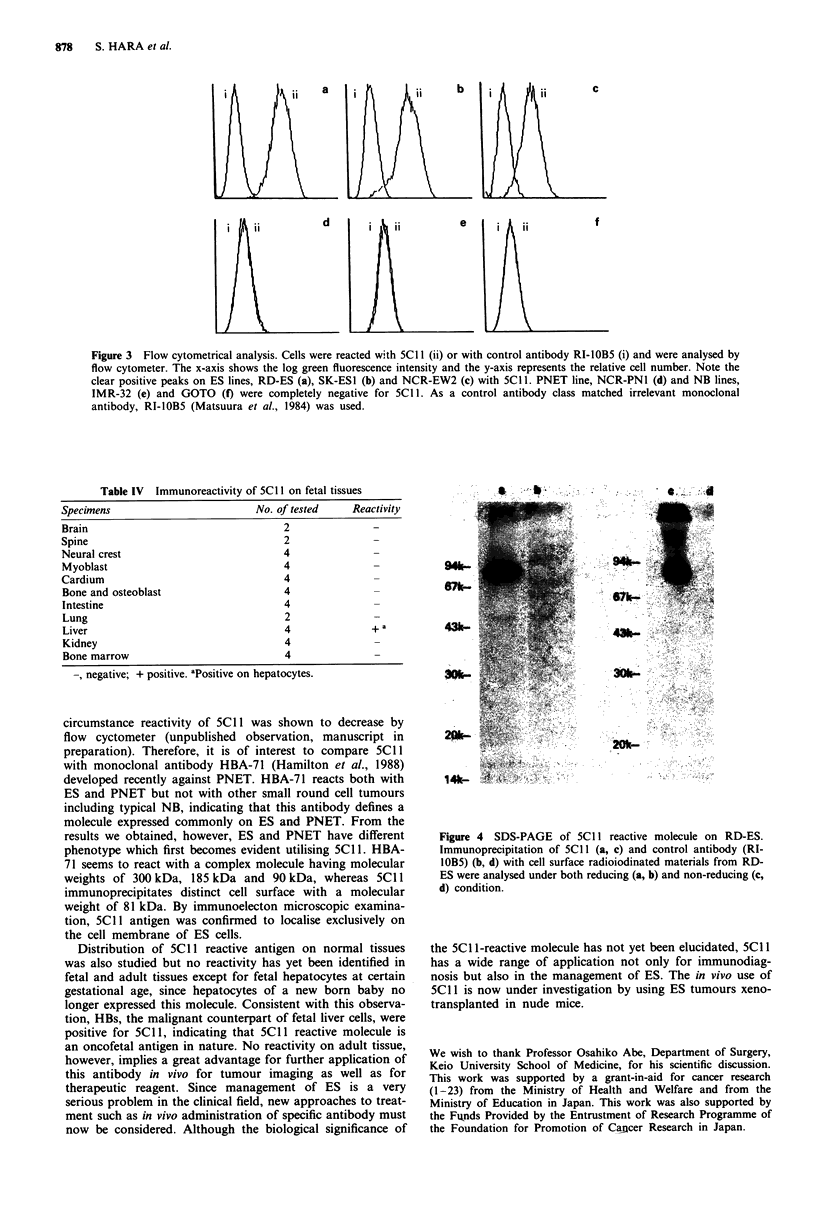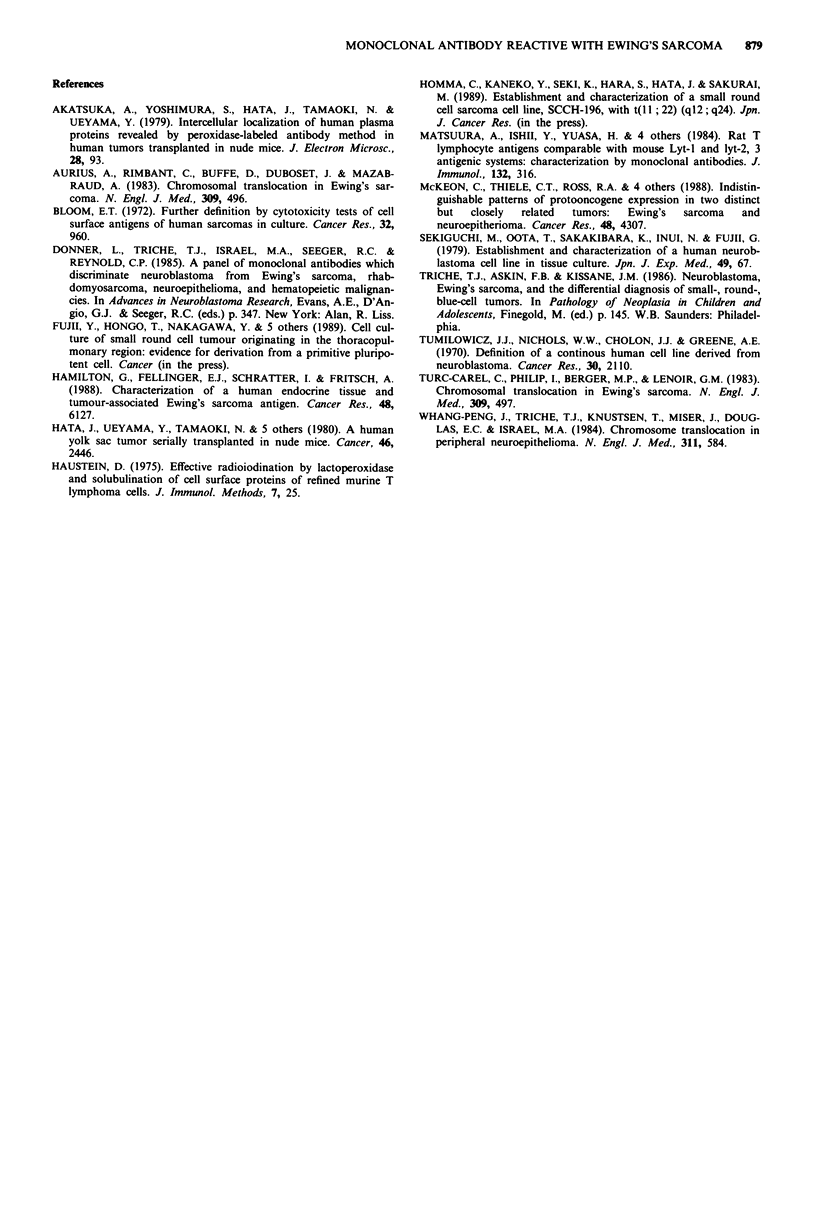# A monoclonal antibody specifically reactive with Ewing's sarcoma.

**DOI:** 10.1038/bjc.1989.383

**Published:** 1989-12

**Authors:** S. Hara, E. Ishii, S. Tanaka, J. Yokoyama, K. Katsumata, J. Fujimoto, J. Hata

**Affiliations:** Department of Pathology, National Children's Medical Research Centre, Tokyo, Japan.

## Abstract

**Images:**


Br. J. Cancer (1989), 60, 875-879 © The Macmillan Press Ltd., 1989

### A monoclonal antibody specifically reactive with Ewing's sarcoma

S. Hara[1,2], E. Ishii[1], S. Tanaka[1], J.Yokoyama[2], K. Katsumata[2], J. Fujimoto[1] & J. Hata[1]

[1]*Department of Pathology, National Children's Medical Research Centre, 3-35-31, Taishido, Setagaya-ku, Tokyo, 154, Japan;* [2]*Department of Surgery, Keio University School of Medicine, 35, Shinano-machi, Shinjuku-ku, Tokyo, 160, Japan.*

**Summary** We have developed a mouse monoclonal antibody 5C11 (IgG2a) against cell surface antigen of Ewing's sarcoma (ES). 5C11 specifically reacted with ESs but not with other small round cell tumours in childhood, i.e. neuroblastomas, primitive neuroectodermal tumours (PNETs), rhabdomyosarcomas and malignant lymphomas. 5C11 did not react with any other tumours in children except for hepatoblastomas. No reactivity has been identified in normal tissues with the exception of fetal hepatocytes. Immunoelectron microscopically, 5C11 reactive antigen was located on cell membrane of ES cells. Biochemically, 5C11 immunoprecipitated a cell surface protein having molecular weight of 81,000 Da. 5C11 is the first antibody which can clearly distinguish ES from neurogenic tumours, especially from PNETs which were recently reported to have common features to ESs regarding chromosal abnormality and proto-oncogene expression but show evident differentiation into neurogenic direction. The results strongly indicate the usefulness of 5C11 not only for diagnostic purpose when no specific marker is available but also for studying the histogenesis of ES. In addition, no reactivity in normal tissue implies its potential application as a therapeutic reagent when the management of ES patients is still a great problem in clinical field.

Ewing's sarcoma (ES) belongs morphologically to the group of small round cell tumours of children because of the absence of unequivocal features of differentiation. This tumour arises from bone and soft tissue, and the histogenesis is still unknown. By electron microscopy, several characteristic features were reported, but specific features of diagnostic value have not been found. Although several monoclonal and polyclonal antibodies were applied to ES, no membrane antigen or protein were known to be specific for ES, and thus the immunodiagnosis of this tumour has been uncertain (Donner *et al.*, 1985; Triche *et al.*, 1986). In this study, we have established a mouse monoclonal antibody, 5C11 which specifically reacts with ES. The unique distribution and partial biochemical characterisation of 5C11-defined molecule are reported.

### Materials and methods

*Development of mouse monoclonal antibodies*

ES cell line RD-ES (obtained from American Type Culture Collection (ATCC)) cells ($5 \times 10^7$ cells) were injected into the intraperitoneal cavity of Balb/cA mice at 2-week intervals three times. Four days after the last boost, immune splenocytes were fused with NS-1 mouse myeloma cells. Supernatants of growing hybridomas were screened on acetone-fixed frozen sections of CR-EW1 tumour, and ES line heterotransplanted in the nude mice by the indirect immunoperoxidase method described below. Hybridomas secreting antibodies of interest were cloned twice by limiting dilution. Immunoglobulin (Ig) subclass monoclonal antibodies were determined by immunodiffusion by rabbit anti-mouse Ig subclass antisera (BIO RAD Laboratories).

*Source of tumours*

Surgically resected human tumours, tumours maintained in a nude mice heterotransplantation system and human cell lines maintained in culture medium were used in this study (Table I). Seventeen osseous and extraosseous ES tumours and cell lines were examined in this study. The transplanted tumour CR-EW2 was derived from case 3. ES cell line NCR-EW1 was derived from CR-EW1, NCR-EW2 from case 2, NCR-EW3 from case 3 and NCR-EW4 from case 5. SCCH-196

**Table I** Ewing's sarcomas examined in this study

| *Case* | *Age (years)/sex* | *Origin* | *O/EO*[a] |
|---|---|---|---|
| Surgical speciments | | | |
| 1 | 7/F | Mediastinum | EO |
| 2 | 13/M | Pelvis | EO |
| 3 | 10/M | Chest wall | EO |
| 4 | 34/M | Chest wall | EO |
| 5 | 14/F | Mediastinum | EO |
| 6 | 9/M | R-femor | O |
| 7 | 14/F | Hip | EO |
| Cell lines | | | |
| NCR-EW1 | 10/M | Neck | EO |
| NCR-EW2 | 13/M | Pelvis | EO |
| NCR-EW3 | 10/M | Chest wall | EO |
| NCR-EW4 | 14/F | Chest wall | EO |
| Transplanted tumours | | | |
| CR-EW1 | 10/M | Neck | EO |
| CR-EW2 | 10/M | Chest wall | EO |

[a]O, osseous; EO, extraosseous.

(Homma *et al.*, 1989) line was kindly provided by Y. Kaneko (Laboratory Medicine, Saitama Cancer Centre Hospital, Saitama) and W-ES line by Y. Fujii (Department of Paediatrics, Hamamatsu University, School of Medicine, Shizuoka; Fujii *et al.*, 1989). Both SCCH-196 and W-ES lines have been shown to have chromosomal translocation with t(11;22). RD-ES and SK-ES1 (Bloom, 1972) were both obtained from ATCC. Three cases of primitive neuroectodermal tumour (PNET)s including one cell line (NCR-PN1) established from a 2-month-old girl with sciatic nerve tumour in our own laboratory, were used in this study. In contrast to ES, these three PNETs revealed ultrastructural and immunohistochemical findings of neurogenic tumours (unpublished data). Briefly, these tumours were shown to have occasional neurosecretory granules and microtubules in the cytoplasmic processes, and they were all positive for anti-neurofilament antibody (200 kDa; Labsystem, Helsinki, Finland) and anti-neuron specific enolase antisera (Dakopatts, California).

These tumour tissues were immediately frozen in OCT-compound (Tissue Tek Division, Miles Scientific Laboratories Inc.) and stored at −80°C until use, or fixed in 20% formaldehyde and embedded in paraffin.

*Preparation of normal and fetal tissues*

Normal tissues were obtained from surgery and autopsy. Normal fetal tissues of 6–12 weeks gestational age which were obtained at the time of necropsy after spontaneous and legal abortion were frozen and stored as described above.

Correspondence: J. Hata.
Received 15 May 1989; and in revised form 25 July 1989.

*Immunostaining of tissues*

Indirect immunoperoxidase staining was performed on acetone-fixed or 4% paraformaldehyde-fixed frozen sections. Slides were incubated with monoclonal antibodies (50 µl of culture supernatant) for 30 min at room temperature in a humidified chamber. After rinsing in 0.01M phosphate buffered saline (PBS), horseradish peroxidase conjugated rabbit IgG anti-mouse whole Igs (Dakopatts, Glostrup, Denmark) were similarly applied on slides for 30 min at room temperature. The slides were then washed in PBS and reacted with diaminobenzidine (2 mg ml$^{-1}$, in 0.5 M Tris-CHl, pH 7.4, and 0.005% $H_2O_2$). The sections were counterstained with methyl green and were observed under light microscopy. When sections of tissues obtained from nude mice were used, endogenous mouse Ig, which would react with the second reagent, were blocked by treating the slides with a Fab fragment of rabbit anti-mouse Ig before application of the first monoclonal antibodies. In some experiments, culture cells were stained by an immunoperoxidase method in the same manner after they were attached to slides by centrifugation using Cytospin (Shandon Southern Products Limited, Cheshire, England).

*Flow cytometrical analysis*

Two million cells were suspended in 100 µl of PBS and incubated with monoclonal antibodies (100 µl of culture supernatant) for 30 min at 4°C. After washing in PBS by centrifugation, they were incubated with fluorescein-conjugated rabbit IgG to mouse Igs (Dakopatts, Glostrup, Denmark). After washing in PBS, they were resuspended in PBS and 10,000 (cells were analysed by flow cytometer (Epics-Profile, Coutler Corporation, Florida, USA)).

*Immunoelectron microscopy*

RD-ES cells were fixed in 0.05 M phosphate-buffered, 4% paraformaldehyde and attached to slides by Cytospin. They were examined by immunoelectron microscopy according to the method previously described (Akatsuka *et al.*, 1979; Hata *et al.*, 1980).

*Immunoprecipitation and SDS-PAGE*

RD-ES cells were externally labelled with $^{125}$I by lactoperoxidase. Briefly, $2 \times 10^7$ cells were labelled with 1 mCi of Na $^{125}$I by the lactoperoxidase catalysing method described by Haustein (1975). After iodination, cells were washed three times with PBS and the cell membrane was disrupted by adding 1 ml of lysing buffer (1% Triton X-100, 0.05 M Tris-HCl, pH 7.4 containing 0.14 M NaCl, 0.05 M EDTA, 0.01 M phenylmetyl sulphonyl fluoride, 0.05% sodium azide) for 1 h at 4°C. After centrifugation, 200 µl of the labelled cell membrane (equivalent to $10^6$ c.p.m.) were incubated with monoclonal antibodies coupled to CNBr-activated Sepharose 4B (Pharmacia) (1 mg antibodies per ml of wet volume) for 3 h at 4°C. The beads were washed with washing buffer (0.01 M Tris-HCl, pH 8.0, 0.5 M NaCl, 0.1% Triton X-100, 0.05% sodium azide), and were extracted with sodium dodesyl sulphate-poly acrylamide gel electrophoresis (SDS-PAGE) sample buffer (0.06 M Tris-HCl, 3% SDS, 10% glycerol, 0.04% bromophenol blue, with and without 2-mercaptoethanol (2-ME)) and boiled for 5 min. The SDS-PAGE was performed either under reducing condition with 5% 2-ME or non-reducing condition without 2-ME. The gel was dried and autoradiographed on Kodak XAR-5 X-ray film.

### Results

*Establishment of mouse monoclonal antibodies*

Of 406 growing hybridomas, three clones were selected because they reacted strongly with ES but not, or weakly, with neuroblasoma (NB) and normal kidney by immunoperoxidase staining. Two clones (7H6 and 8B1) reacted with both ES and NB, but also with some components of kidney. On the other hand, 5C11 only reacted with Es but not with NB and kidney. In this study, 5C11 was further investigated. The Ig subclass of 5C11 was determined as IgG2a by immunodiffusion.

*Reactivity of 5C11 on human tumours*

Reactivity of 5C11 with various human tumours was investigated by immunoperoxidase and the results are listed in Table II. 5C11 reacted with 16 of 17 ESs regardless of their origins (osseous or extraosseous). Figure 1a and b shows the typical histology of ES. In Figure 1b, a rosette-like structure is clearly seen. As is shown in Figure 1c, 5C11 reacts with all tumour cells of ES. One ES line, NCR-EW1, unreactive to 5C11, was derived from the transplanted tumour CR-EW1 which was used at the first screening as positive control. Although the mechanism is unknown, 5C11 expression seemed to be lost during *in vitro* culture in this particular cell line. The localisation of 5C11 antigen on ES cells was further investigated by immunoelectron microscopy and the antigen was found to be present on the cell membrane (Figure 2). 5C11 did not react with NBs, PNETs, rhabdomyosarcomas and lymphomas. Identical results were obtained on cell ines by immunofluorescence followed by flow cytometrical analysis. Typical examples are shown in Figure 3. Thus ES lines RD-ES, SK-ES1, NCR-EW2 (Figure 3a, and c, respectively) were all 5C11 positive, whereas PNET line (NCR-PN1) (Figure 3d) and NB lines, IMR-32 (Tumilowicz *et al.*, 1970) (Figure 3e) and GOTO (Sekiguchi *et al.*, 1979 and Figure 3f) were completely 5C11 negative. 5C11 was investigated on other human tumours in children and it was found that 5C11 did not react with any other tumours except for hepatoblastomas (HB). On HBs 5C11 reacted with all four cases. The reactivity of 5C11 on HB was found on cell surface. Reactivity of 5C11 was stable on acetone-fixed frozen section and paraformaldehyde-fixe tissue but was lost when tissues were processed for formalin-fixation followed by paraffin-embedding.

*Reactivity of 5C11 on normal and fetal tissues*

A variety of normal and fetal human tissues were studied for the distribution of 5C11-defined antigen. 5C11 did not react with any normal tissues with the number of samples indicated (Table III). In fetal tissues, a strong reactivity of 5C11 was detected in hepatocytes but no reactivity was found in any other tissues (Table IV). Interestingly, 5C11 did not react with hepatocytes of new born babies and adults, indicating the oncofetal nature of 5C11-defined antigen.

*Biochemical characterisation of 5C11-defined molecule*

Biochemical characterisation of 5C11-defined molecule was carried out using RD-ES line. As is shown in Figure 4, 5C11 immunoprecipitated a protein having molecular weight (MW)

**Table II** Immunoreactivities of 5C11 on tumours

| *Tumour tissues tested* | *No. of positive / No. tested* |
|---|---|
| Ewing's sarcoma | |
| EO[a] | 13/14 |
| O[a] | 2/2 |
| unknown | 1/1 |
| Neuroblastoma | 0/22 |
| PNET | 0/3 |
| Rhabdomyosarcoma | 0/10 |
| Osteosarcoma | 0/3 |
| Wilm's tumour | 0/5 |
| Hepatoblastoma | 4/4 |
| Malignant lymphoma | 0/3 |

PNET, primitive neuroectodermal tumour. [a]EO, extraosseous; O, osseous.

**Figure 1** Histology of ESs (paraffin section) and the reactivity of 5C11 with these tumour (frozen section). **a** (case 2), **b** (case 3), Haematoxylin and Eosin stain ( × 100). **b**, rosette-like arrangement is clearly seen in case 3. **c**, 5C11 stain ( × 100) on case 3. 5C11 reacts with all tumour cells in case 3. Identical reactivity was observed in case 2 (data not shown). R, rosette-like arrangement. **d**, control antibody.

of 81 KD (Figure 4 lanes a and c) under both reducing and non-reducing conditions, suggesting that 5C11 reactive moleclues do not have a subunit structure linked by a disulphide bond. No specific band was identified when class matched irrelevant antibody was used as control (Figure 4 lanes b and d).

5C11 reactive antigen on HB was also studied using cell suspension prepared from heterotransplantable HB, CR-HP3 and it was found that 5C11 reacted with the protein having a molecular weight identical to that on ES (data not shown).

### Discussion

Among malignant small round cell tumours, ES is considered as the most primitive due to the absence of unequivocal morphological characteristics as well as differentiation capability. Although several hypotheses have been reported, the histogenesis of ES still remains obscure. Accordingly, the specific features of diagnostic value of this tumour have not yet been determined.

**Figure 2** Immunoelectron microscopic detection of 5C11-defined antigen in RD-ES cell. Note the location of 5C11 reactive molecule on the cell membrane ( × 3700). N, nucleus of the tumour cell.

In an effort to develop a reliable probe useful for characterising ES, we have successfully established a monoclonal antibody 5C11 which specifically reacts with ES. Reactivity of 5C11 was examined with a panel of tumour tissues and it was found that 5C11 only reacted with ES among tumours in childhood except for HB. In addition, 5C11 reacted with both osseous and extraosseous ESs. Identical specificity of 5C11 was also shown on a variety of cell lines *in vitro*. These results clearly indicate that 5C11 is an extremely useful tool in diagnostic pathology field for prompt and accurate diagnosis. It is of note that 5C11 can distinguish ES from PNET, the latter of which shows more clear differentiation capability towards neurogenic tissues as revealed by ultrastructural features such as the presence of neurosecretory granules and by the production of tissue specific proteins such as neurofilaments. On the other hand, cytogenetical analysis has shown that PNET and some ES have the same chromosomal abnormalities involving (11 ; 22) (q24 ; q12) (Aurius *et al.*, 1983; Turc-Carel *et al.*, 1983; Whang-Peng *et al.*, 1984). In addition, common proto-oncogene expression has been observed in PNET and ES (Mckeon *et al.*, 1988). In fact, some ES cells which we established could be induced to differentiate into neural direction by the administration of cyclic AMP *in vitro* (unpublished observation). In such a

**Table III** Immunoreactivity of 5C11 on normal tissues

| *Specimens* | *No. of tested* | *Reactivity* |
|---|---|---|
| Brain | 5 | – |
| Spine | 5 | – |
| Stomach | 4 | – |
| Intestine | 4 | – |
| Lung | 4 | – |
| Liver | 5 | – |
| Kidney | 5 | – |
| Adrenal cortex | 6 | – |
| Adrenal medulla | 6 | – |
| Sympathetic ganglion | 4 | – |
| Testis | 4 | – |
| Muscle | 4 | – |
| Bone | 4 | – |
| Bone marrow | 8 | – |
| Peripheral blood | 4 | – |
| Thymus | 4 | – |
| Tonsil | 4 | – |
| Lymph node | 5 | – |

–, negative; + positive.

**Figure 3** Flow cytometrical analysis. Cells were reacted with 5C11 (ii) or with control antibody RI-10B5 (i) and were analysed by flow cytometer. The x-axis shows the log green fluorescence intensity and the y-axis represents the relative cell number. Note the clear positive peaks on ES lines, RD-ES (**a**), SK-ES1 (**b**) and NCR-EW2 (**c**) with 5C11. PNET line, NCR-PN1 (**d**) and NB lines, IMR-32 (**e**) and GOTO (**f**) were completely negative for 5C11. As a control antibody class matched irrelevant monoclonal antibody, RI-10B5 (Matsuura et al., 1984) was used.

**Table IV** Immunoreactivity of 5C11 on fetal tissues

| *Specimens* | *No. of tested* | *Reactivity* |
|---|---|---|
| Brain | 2 | – |
| Spine | 2 | – |
| Neural crest | 4 | – |
| Myoblast | 4 | – |
| Cardium | 4 | – |
| Bone and osteoblast | 4 | – |
| Intestine | 4 | – |
| Lung | 2 | – |
| Liver | 4 | +[a] |
| Kidney | 4 | – |
| Bone marrow | 4 | – |

–, negative; + positive. [a]Positive on hepatocytes.

circumstance reactivity of 5C11 was shown to decrease by flow cyctometer (unpublished observation, manuscript in preparation). Therefore, it is of interest to compare 5C11 with monoclonal antibody HBA-71 (Hamilton et al., 1988) developed recently against PNET. HBA-71 reacts both with ES and PNET but not with other small round cell tumours including typical NB, indicating that this antibody defines a molecule expressed commonly on ES and PNET. From the results we obtained, however, ES and PNET have different phenotype which first becomes evident utilising 5C11. HBA-71 seems to react with a complex molecule having molecular weights of 300 kDa, 185 kDa and 90 kDa, whereas 5C11 immunoprecipitates distinct cell surface with a molecular weight of 81 kDa. By immunoelecton microscopic examination, 5C11 antigen was confirmed to localise exclusively on the cell membrane of ES cells.

Distribution of 5C11 reactive antigen on normal tissues was also studied but no reactivity has yet been identified in fetal and adult tissues except for fetal hepatocytes at certain gestational age, since hepatocytes of a new born baby no longer expressed this molecule. Consistent with this observation, HBs, the malignant counterpart of fetal liver cells, were positive for 5C11, indicating that 5C11 reactive molecule is an oncofetal antigen in nature. No reactivity on adult tissue, however, implies a great advantage for further application of this antibody *in vivo* for tumour imaging as well as for therapeutic reagent. Since management of ES is a very serious problem in the clinical field, new approaches to treatment such as *in vivo* administration of specific antibody must now be considered. Although the biological significance of

**Figure 4** SDS-PAGE of 5C11 reactive molecule on RD-ES. Immunoprecipitation of 5C11 (**a, c**) and control antibody (RI-10B5) (**b, d**) with cell surface radioiodinated materials from RD-ES were analysed under both reducing (**a, b**) and non-reducing (**c, d**) condition.

the 5C11-reactive molecule has not yet been elucidated, 5C11 has a wide range of application not only for immunodiagnosis but also in the management of ES. The *in vivo* use of 5C11 is now under investigation by using ES tumours xenotransplanted in nude mice.

We wish to thank Professor Osahiko Abe, Department of Surgery, Keio University School of Medicine, for his scientific discussion. This work was supported by a grant-in-aid for cancer research (1–23) from the Ministry of Health and Welfare and from the Ministry of Education in Japan. This work was also supported by the Funds Provided by the Entrustment of Research Programme of the Foundation for Promotion of Cancer Research in Japan.

### References

AKATSUKA, A., YOSHIMURA, S., HATA, J., TAMAOKI, N. & UEYAMA, Y. (1979). Intercellular localization of human plasma proteins revealed by peroxidase-labeled antibody method in human tumors transplanted in nude mice. *J. Electron Microsc.*, **28**, 93.

AURIUS, A., RIMBANT, C., BUFFE, D., DUBOSET, J. & MAZABRAUD, A. (1983). Chromosomal translocation in Ewing's sarcoma. *N. Engl. J. Med.*, **309**, 496.

BLOOM, E.T. (1972). Further definition by cytotoxicity tests of cell surface antigens of human sarcomas in culture. *Cancer Res.*, **32**, 960.

DONNER, L., TRICHE, T.J., ISRAEL, M.A., SEEGER, R.C. & REYNOLD, C.P. (1985). A panel of monoclonal antibodies which discriminate neuroblastoma from Ewing's sarcoma, rhabdomyosarcoma, neuroepithelioma, and hematopeietic malignancies. In *Advances in Neuroblastoma Research*, Evans, A.E., D'Angio, G.J. & Seeger, R.C. (eds.) p. 347. New York: Alan, R. Liss.

FUJII, Y., HONGO, T., NAKAGAWA, Y. & 5 others (1989). Cell culture of small round cell tumour originating in the thoracopulmonary region: evidence for derivation from a primitive pluripotent cell. *Cancer* (in the press).

HAMILTON, G., FELLINGER, E.J., SCHRATTER, I. & FRITSCH, A. (1988). Characterization of a human endocrine tissue and tumour-associated Ewing's sarcoma antigen. *Cancer Res.*, **48**, 6127.

HATA, J., UEYAMA, Y., TAMAOKI, N. & 5 others (1980). A human yolk sac tumor serially transplanted in nude mice. *Cancer*, **46**, 2446.

HAUSTEIN, D. (1975). Effective radioiodination by lactoperoxidase and solubulination of cell surface proteins of refined murine T lymphoma cells. *J. Immunol. Methods*, **7**, 25.

HOMMA, C., KANEKO, Y., SEKI, K., HARA, S., HATA, J. & SAKURAI, M. (1989). Establishment and characterization of a small round cell sarcoma cell line, SCCH-196, with t(11 ; 22) (q12 ; q24). *Jpn. J. Cancer Res.* (in the press).

MATSUURA, A., ISHII, Y., YUASA, H. & 4 others (1984). Rat T lymphocyte antigens comparable with mouse Lyt-1 and lyt-2, 3 antigenic systems: characterization by monoclonal antibodies. *J. Immunol.*, **132**, 316.

McKEON, C., THIELE, C.T., ROSS, R.A. & 4 others (1988). Indistinguishable patterns of protooncogene expression in two distinct but closely related tumors: Ewing's sarcoma and neuroepitherioma. *Cancer Res.*, **48**, 4307.

SEKIGUCHI, M., OOTA, T., SAKAKIBARA, K., INUI, N. & FUJII, G. (1979). Establishment and characterization of a human neuroblastoma cell line in tissue culture. *Jpn. J. Exp. Med.*, **49**, 67.

TRICHE, T.J., ASKIN, F.B. & KISSANE, J.M. (1986). Neuroblastoma, Ewing's sarcoma, and the differential diagnosis of small-, round-, blue-cell tumors. In *Pathology of Neoplasia in Children and Adolescents*, Finegold, M. (ed.) p. 145. W.B. Saunders: Philadelphia.

TUMILOWICZ, J.J., NICHOLS, W.W., CHOLON, J.J. & GREENE, A.E. (1970). Definition of a continous human cell line derived from neuroblastoma. *Cancer Res.*, **30**, 2110.

TURC-CAREL, C., PHILIP, I., BERGER, M.P., & LENOIR, G.M. (1983). Chromosomal translocation in Ewing's sarcoma. *N. Engl. J. Med.*, **309**, 497.

WHANG-PENG, J., TRICHE, T.J., KNUSTSEN, T., MISER, J., DOUGLAS, E.C. & ISRAEL, M.A. (1984). Chromosome translocation in peripheral neuroepithelioma. *N. Engl. J. Med.*, **311**, 584.